# Supplementary material for: Chronic Intestinal Failure in Children: An International Multicenter Cross-Sectional Survey
Source: Nutrients. 2022 Apr 30;14(9):1889. doi: 10.3390/nu14091889 (PMC9103944; doi:10.3390/nu14091889)
Supplement: Supplementary file 1 [file nutrients-14-01889-s001.zip › nutrients-1668035-supplementary.pdf]

Supplementary Table S1. Contributing home parenteral nutrition (HPN) centers and patients with chronic intestinal failure due to benign disease enrolled in the study, grouped by country of origin.

	HPN Centers	Patients	
**Country**	**n.**	**n.**	**%**
Italy	13	204	36.6%
France	4	78	14.0%
The Netherlands	3	69	12.4%
United Kingdom	7	55	9.9%
Israel	2	33	5.9%
Australia	4	31	5.6%
Finland	1	27	4.9%
Croatia	1	16	2.9%
Belgium	1	9	1.6%
Bulgaria	1	6	1.0%
Spain	3	6	1.0%
Sweden	1	6	1.0%
Argentina	1	5	0.9%
Lithuania	1	5	0.9%
Denmark	2	4	0.7%
Others	3	4	0.7%
Total	49	558	100.0%

Others: Brazil, US.

Supplementary Table S2. Comparison of nutritional status parameters (z-scores) for total cohort versus cohort after excluding children with necrotizing enterocolitis (NEC) aged <2 years at inclusion in the database.

	**Total cohort (n. 558)**	**NEC aged <2 years excluded (n. 525)**
***WFA z-score**, median*	-1.15	-1.15
z-score category		
< -2	27.5%	26.7%
-2 – -1	25.1%	26.3%
-1 – 1	41.3%	40.9%
1 – 2	4.6%	4.7%
> 2	1.5%	1.4%
***LFA/HFA z-score**, median (IQR)*	-1.15	-1.13
z-score category		
< -2	30.2%	29.5%
-2 – -1	23.7%	24.0%
-1 – 1	39.0%	39.5%
1 – 2	4.0%	4.1%
> 2	3.1%	2.9%
***BMI-FA** z-score, median (IQR)*	-0.38	-0.40

z-score category		
< -2	15.1%	15.3%
-2 - -1	19.2%	19.4%
-1 - 1	52.1%	52.0%
1 - 2	10.7%	10.2%
> 2	2.9%	3.1%

Statistic: no significant differences between the 2 groups. Abbreviations: WFA, body weight for age; LFA/HFA, body length for age (patients aged 0-2 years) or height for age (patients aged >2 years); BMI-FA, body mass index for age.

Supplementary Table S3. Analysis of factors associated with the nutritional status parameters (z-scores).

		**WFA z-score**			
		**Univariate analysis**		**Comparison between categories**	
	**n.**	**rho**	**p**	**Medians (IQR)**	p
**Age,** yr	545	-0.093	**0.030**		
**Category**					
<1	75			-1.6 (2.6)	0.002
1-4	178			-0.8 (1.9)	0.010
4-10	131			-0.6 (1.6)	<0.001
10-14	64			-1.3 (1.9)	
14-18	97			-1.7 (2.0)	
**Age at starting HPN**, yr	449	-0.057	0.231		
**Category**					
<1	257			-1.0 (1.9)	0.024
1-4	85			-0.7 (1.7)	0.025
4-10	45			-0.6 (1.3)	0.014
10-14	27			-1.9 (2.4)	
14-18	35			-2.1 (2.2)	
**HPN duration,** mo	530	0.064	0.142		
**Category**					
<12	219			-1.4 (2.4)	0.010
12-24	72			-0.5 (1.8)	
24-36	47			-0.8 (1.8)	0.003
36-60	62			-0.4 (2.0)	0.025
60-120	82			-0.9 (1.2)	
>120	48			-1.7 (1.4)	
**IVS energy** (%IVSE/REE)	527	-0.096	**0.027**		
**Category**					
≤50%	61			-0.8 (1.8)	
50%-75%	76			-1.0 (1.8)	
75%-100%	84			-1.2 (2.4)	
100%-125%	114			-0.8 (1.8)	

125%-150%	108			-1.0 (2.0)	
≥150%	84			-1.4 (2.2)	
**Sex**			0.600		
Males	319			-1.1 (2.0)	
Females	239			-1.0 (1.9)	
**Mechanism of CIF**					
SBS-J	77		0.520	-1.1 (2.4)	
SBS-JC	103		**0.009**	-0.6 (1.8)	0.005
SBS-JIC	77		0.143	-0.8 (1.6)	0.044
Dysm	144		0.652	-1.1 (2.2)	
MD	112		**0.006**	-1.5 (1.9)	

		**LFA/HFA z-score**			
		**Univariate analysis**		**Comparison between categories**	
	**n.**	**rho**	**p**	**Medians (IQR)**	**p**
**Age,** yr	523	-0.037	0.393		
**Category**					
<1	72			-1.0 (3.7)	
1-4	167			-1.0 (2.3)	
4-10	128			-1.1 (1.8)	
10-14	64			-1.6 (2.5)	
14-18	92			-1.1 (1.6)	
**Age at starting HPN**, yr	429	0.007	0.890		
**Category**					
<1	249			-1.1 (2.4)	
1-4	78			-1.1 (2.2)	
4-10	43			-1.0 (1.6)	
10-14	27			-1.2 (2.9)	
14-18	32			-0.9 (1.4)	
**HPN duration,** mo	510	-0.097	**0.049**		
**Category**					
<12	211			-1.1 (2.4)	<0.001
12-24	65			-0.7 (1.6)	<0.001
24-36	46			-1.1 (2.2)	0.003
36-60	59			-0.6 (1.9)	0.001
60-120	82			-1.3 (1.8)	0.041
>120	47			-2.0 (1.5)	

**IVS energy** (%IVSE/REE)	511	-0.076	**0.085**		
**Category**					
≤50%	58			-1.2 (2.2)	
50%-75%	76			-1.0 (1.7)	
75%-100%	81			-1.0 (2.1)	
100%-125%	106			-0.9 (2.0)	
125%-150%	107			-1.3 (2.1)	
≥150%	83			-1.4 (2.9	
**Sex**			0.886		
Males	319			-1.1 (2.0)	
Females	239			-1.1 (2.2)	
**Mechanism of CIF**					
SBS-J	76		0.590	-1.3 (2.0)	
SBS-JC	100		**0.059**	-0.9 (1.7)	0.001
SBS-JIC	74		0.321	-1.0 (1.7)	0.010
Dysm	137		**0.056**	-0.9 (1.8)	0.001
MD	106		**<0001**	-1.9 (2.2)	

		**BMI-FA z-score**			
		**Univariate analysis**		**Comparison between categories**	
	**n.**	**rho**	**p**	**Medians (IQR)**	p
**Age,** yr	522	-0.078	**0.076**		
**Category**					
<1	72			-0.9 (2.0)	
1-4	167			-0.1 (1.9)	
4-10	127			-0.0 (1.5)	All <0.001
10-14	64			-0.4 (1.2)	
14-18	92			-0.9 (2.0)	
**Age at starting HPN**, yr	429	-0.063	0.191		
**Category**					
<1	249			-0.3 (1.8)	
1-4	78			0.0 (1.5)	
4-10	43			-0.1 (1.6)	All <0.001
10-14	27			-0.7 (2.3)	
14-18	32			-1.9 (2.6)	
**HPN duration,** mo	509	0.158	**<0.001**		
**Category**					
<12	211			-0.8 (2.1)	0.034
12-24	65			-0.0 (1.9)	
24-36	46			-0.3 (1.7)	0.020
36-60	59			0.1 (2.2)	

60-120	81			-0.3 (1.1)	
>120	47			-0.2 (1.9)	
**IVS energy** (%IVSE/REE)	510	0.059	0.180		
**Category**					
≤50%	58			0.0 (1.7)	
50%-75%	75			-0.3 (1.9)	
75%-100%	81			-0.6 (1.9)	
100%-125%	106			-0.4 (1.6)	
125%-150%	107			-0.1 (1.7)	
≥150%	83			-0.5 (2.0)	
**Sex**			0.265		
Males	319			-0.4 (2.0)	
Females	239			-0.2 (1.5)	
**Mechanism of CIF**					
SBS-J	75		0.691	-0.3 (2.2)	
SBS-JC	100		0.427	-0.3 (1.9)	
SBS-JIC	74		0.759	-0.4 (1.6)	
Dysm	137		**0.099**	-0.4 (2.2)	
MD	106		0.270	-0.3 (2.1)	

Statistic: Univariate analysis by Spearman's test was used for the continuous variables and by Mann-Whitney's test for the binary variable sex; the categorical variable "mechanism of CIF" was dichotomized and each dummy variable was analyzed individually.The relative effects between the categories of the ordinal variables were compared using the nonparametric multiple comparisons for relative effects test with Tukey's test for the contrasts.

Abbreviations: WFA, body weight for age; LFA/HFA, body length for age (patients aged 0-2 years) or height for age (patients aged >2 years); BMI-FA, body mass index for age; IVS energy (%IVSE/REE), ratio of daily intravenous supplementation total energy over estimated resting energy expenditure; HPN, home parenteral nutrition; : CIF, chronic intestinal failure; SBS-J, SBS with end jejunostomy or ileostomy (included also enterocutaneous fistulas, n.7); SBS-JC, jejunocolic anastomosis; SBS-JIC, jejunoileal anastomosis with an intact colon and ileocecal valve; Dysm, dysmotility (included also patients with mechanical obstruction, n. 8); MD, mucosal disease.

Supplementary Table S4. Analysis of factors associated with IVS energy (%IVSE/REE).

	IVS energy (%IVSE/REE)				
	Univariate analysis			Comparison between categories	
	n.	rho	p	Medians (IQR)	p
**Age,** yr	532	-0.097	**0.025**		
**Category**					
<1	73			131.8 (80.8)	
1-4	170			106.0 (68.1)	
4-10	128			109.0 (55.3)	

10-14	64			114.8) (52.5)	0.003	0.010
					0.007	
14-18	97			103.4 (55.7)		
**Age at starting**, yr	434	-0.104	**0.031**			
**Category**						
<1	250			113.4 (73.4)		
1-4	80			115.1 (53.4)		
4-10	44			107.1 (38.9)		
10-14	27			103.4 (98.1)		
14-18	33			97.6 (43.5)		
**HPN duration,** mo	516	-0.104	0.825			
**Category**						
<12	213			111.1 (68.6)		
12-24	63			113.3 (58.3)		
24-36	46			89.6 (69.4)		
36-60	62			108.0 (54.0)		
60-120	83			113.5 (66.4)		
>120	49			118.2 (55.7)		
**Sex**			**0.060**			
Males	302			107.5 (60.1)		
Females	230			116 (60.4)		
**Mechanism of CIF**						
SBS-J	77		0.296	116.3 (72.6)		
SBS-JC	103		**0.011**	101.8 (60.6)		0.002
SBS-JIC	76		**0.015**	89.5 (63.0)		0.002
Dysm	137		**0.001**	122.1 (57.8)		
MD	108		0.968	111.4 (57.7)		

Statistic: Univariate analysis by Spearman's test was used for the continuous variables and by Mann-Whitney's test for the binary variable sex; the categorical variable "mechanism of CIF" was dichotomized and each dummy variable was analyzed individually.The relative effects between the categories of the ordinal variables were compared using the nonparametric multiple comparisons for relative effects test with Tukey's test for the contrasts.

Abbreviations: IVS energy (%IVSE/REE), ratio of daily intravenous supplementation total energy over estimated resting energy expenditure; HPN, home parenteral nutrition; : CIF, chronic intestinal failure; SBS-J, SBS with end jejunostomy or ileostomy (included also enterocutaneous fistulas, n.7); SBS-JC, jejuno-colic anastomosis; SBS-JIC, jejunoileal anastomosis with an intact colon and ileocecal valve; Dysm, dysmotility (included also patients with mechanical obstruction, n. 8); MD, mucosal disease.
